# Evaluation of the National Competence Based Catalogue of Learning Objectives (NKLM 2.0) for undergraduate medical education at the Medical School Hannover

**DOI:** 10.3205/zma001650

**Published:** 2023-11-15

**Authors:** Marie Mikuteit, Ingo Just, Sandra Steffens

**Affiliations:** 1 Hannover Medical School, Dean’s Office, Hannover, Germany

**Keywords:** National Competence Based Catalogue of Learning Objectives for Undergraduate Medical Education (NKLM), evaluation, phase one, phase two, teaching, curriculum

## Abstract

**Background:**

The National Competence-Based Catalogue of Learning Objectives for Undergraduate Medical Education (NKLM) serves as the foundation for curricular development in undergraduate medical education in Germany. A new version of the NKLM was launched in 2021, and medical faculties are now evaluating the learning objectives (LOs). This paper describes the evaluation process used at Hannover Medical School.

**Methods:**

The evaluation process was structured in three steps. LOs were rated as “keep”, “modify” or “delete”. First, the 1133 LOs were compared with the mapping of the Hannover curriculum from 2017. Then, a small team from the Curricular Development Department conducted a pre-evaluation of the 1133 LOs. Finally, a group of clinical experts and students discussed and agreed on the ratings.

**Results:**

For 868 LOs, one or more counterparts were found in the mapping, but 265 new LOs were not found and thus, classified as new. In the first rating, 779 LOs were kept, 300 were modified (172 due to wording), 45 were deleted, and there was no rating for 9 LOs. The expert group changed 47 of the pre-evaluation decisions. The final rating was to keep 738 LOs, modify 356, and delete 39 LOs.

**Conclusion:**

This method effectively evaluated the LOs from NKLM 2.0 while balancing expert knowledge and an overview of the curriculum.

## Zusammenfassung

**Hintergrund:**

Der Nationale Kompetenzbasierte Lernzielkatalog Medizin (NKLM) dient als Grundlage für die curriculare Lehre der medizinischen Ausbildung in Deutschland. Die neue Version des NKLM 2.0 wurde 2021 eingeführt und medizinische Fakultäten evaluieren nun die Lernziele. Dieser Artikel beschreibt den Evaluationsprozess an der Medizinischen Hochschule Hannover (MHH).

**Methoden:**

Der Evaluationsprozess war in drei Schritte strukturiert. Die Lernziele wurden mit „unverändert erhalten“, „Inhalt anpassen“ oder „komplett streichen“ bewertet. Zuerst wurden die 1133 Lernziele mit dem NKLM Mapping der MHH von 2017 abgeglichen. Dann führte ein kleines Team aus dem Bereich der Curriculumentwicklung eine Vor-Evaluation der 1133 Lernziele durch. Anschließend entschied eine Gruppe von klinischen Expert*innen und Studierenden über die Bewertungen.

**Ergebnisse:**

Bei 868 Lernzielen wurden ein oder mehrere Äquivalente im NKLM Mapping gefunden, wohingegen 265 Lernziele noch nicht vorhanden waren und entsprechend als neu eingestuft wurden. In der ersten Bewertung wurden 779 Lernziele beibehalten, 300 wurden modifiziert (davon 172 aufgrund der Sprache oder Formulierung), 45 wurden verworfen und für 9 Lernziele erfolgte keine Bewertung. Die Expert*innengruppe änderte 47 der Vor-Evaluationsentscheidungen. Die endgültige Bewertung lautete, 738 Lernziele zu erhalten, 356 zu modifizieren und 39 zu verwerfen.

**Schlussfolgerung:**

Diese Methode gestaltete die Bewertung der Lernziele des NKLM 2.0 effektiv unter Berücksichtigung von Expert*innenwissen und einem Überblick über das Curriculum.

## Introduction

In April 2021, a new version of the National Competence-Based Catalogue of Learning Objectives for Undergraduate Medical Education (NKLM 2.0) was released [https://nklm.de/zend/menu]. The development process was led by the Medizinischer Fakultätentag (MFT) [1] and involved experts from medical schools university medicine, research, and various medical societies. The development was based on the LOOOP interactive platform [2], which was used by expert groups to discuss learning objectives (LOs) in different specialties.

The NKLM 2.0 will serve as the basis for medical curricula in the new German Medical practice regulations (ApprO), which is currently under radical revision and is set to be approved in 2027 [3]. The current “Gegenstandskatalog” defines the subjects of national medical exams, whereas the NKLM comprises practical skills and medical competencies in subjects such as collaboration and communication, leadership or science [4]. In the future, the NKLM should define what is taught and tested in medical education. The competence level (factual knowledge, know-how, action competence) and proposed semester for teaching each LO are stated in the NKLM. The catalog can be applied to map the entire curriculum, modify or conceptualize novel courses [5]. Medical educators recognize the relevance and the chances of a national LO catalogue but application is difficult to realize [6].

The MFT is overseeing the improvement process for the current NKLM 2.0. Editing of the 2814 LOs has been divided up into four phases, with 1133 LOs in phases 1 and 2 combined. The medical faculties suggested to divide the process into four phases, so that each phase comprises a manageable amount of LOs. During these editing phases, the faculties evaluate the LOs and decide whether to keep, modify, or delete them. There is no designated evaluation procedure, but regular online meetings with the MFT and the faculties accompany the process, during which different evaluation methods are discussed. In the rating process, expert knowledge of the different medical disciplines and knowledge of the entire medical curriculum must be balanced. The evaluations by the faculties will be processed by expert groups who will create a 2.1 version of the NKLM.

In this paper, we present the evaluation model used for the learning objectives of phases 1 and 2 of the NKLM 2.0 at Hannover Medical School.

## Methods

**Figure 1:**
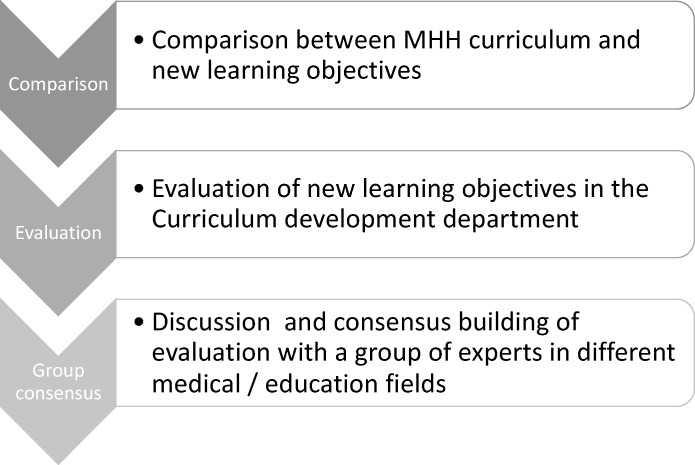
Three steps of the evaluation process of NKLM 2.0 at Hannover medical School. The comparison is based on the mapping process of the NKLM 1.0 from 2017 at MHH. A LO was counted as “taught”, if at least one module stated to teach it explicitly.

The evaluation process for the learning objectives of combined phase 1 and 2 of the National Competence Based Catalogue of Learning Objectives for Undergraduate Medical Education (NKLM 2.0) was operated by the responsible persons in charge of the NKLM and curriculum development at Hannover Medical School in collaboration with the faculty. The process comprises three steps, as illustrated in figure 1 (Fig. 1). The possible ratings focusing on the text of the learning objectives were “keep”, “modify” or “delete”. Modifications involve alterations in wording, removal of interconnections with other learning objectives, splitting of the learning objective into sub-objectives, or aggregation of detailed objectives into higher-level one. Learning objectives covering specialist knowledge or belonging to the elective field have to be deleted.

In the first step, 1133 learning objectives of phases 1 and 2 were matched one-by-one with the mapping results of the NKLM 1.0 [7]. The mapping was performed in 2017 at MHH and each module of the medical course stated which LO are taught. A LO was counted as “taught”, if at least one module stated to teach it explicitly in the mapping of 2017. For each learning objective from the NKLM 2.0, the LOOOP platform provided information on its status compared to the NKLM 1.0. This information indicates that the objective was either a novel one, based on one or more old objectives, or a direct takeover from the NKLM 1.0. For all objectives with a counterpart in NKLM 1.0, additional information was collected on which modules the objective was taught and at which competence level. Since the NKLM 1.0 did not provide any interconnections, we focused on the text of the LOs.

Next, the responsible persons at Hannover Medical School conducted a first rating of the learning objectives of phases 1 and 2, assigning a rating of “keep”, “modify”, or “delete”. Learning objectives that were taught in more than one module according to the mapping from 2017 and novel objectives with useful additions were kept. The objectives were checked for redundancies across all chapters and, if necessary, merged. Objectives that were too detailed were put together with other objectives. The wording was stringently adapted to gender-sensitive language or to better match the competence level of the objective. The competence level was also checked if it was deemed too high or low, and interconnections were adjusted accordingly. Objectives that were redundant, not taught according to the mapping from 2017, or assed irrelevant (such as specialty knowledge) were deleted.

In the final step, the critical learning objectives were discussed with a group of experts from various medical disciplines and students. The group comprised 18 experts and five students covering disciplines such as anesthesia, pediatrics, radiology, biochemistry, gynecology, general practice, pharmacology, medical informatics, rehabilitation, dermatology, surgery, medical psychology, and rheumatology. All new objectives, regardless of the initial rating, were discussed. Additionally, objectives with a rating of “delete” were also discussed. The criteria used in the rating and discussion are displayed in table 1 (Tab. 1).

## Results

Three people were involved in the first two stages of evaluating the NKLM 2.0 at Hannover Medical School, each working for a total of 20 hours. An expert discussion was held with 18 participants, and 30 hours were spent in total preparing and verifying the results.

In the first step, the new LOs were compared to those of the NKLM 1.0. All LOs from the NKLM 2.0 had at least one equivalent LO in the NKLM 1.0 that was covered by modules at MHH according to the 2017 mapping. The majority of LOs were taught by more than one module. 264 LOs of NKLM 2.0 had no equivalent in NKLM 1.0 and were thus classified as new. 

In the second step, the NKLM team performed a pre-rating. Of the total of 1133 LOs, 778 (68.7%) were rated as to be “kept”, 300 (26.5%) as to be modified (of which 172 (57.3%) were “modified” because of the wording), and 45 (3.9%) were rated as to be “deleted”. Most LOs were changed due to wording issues or were merged with similar LOs. Nine (0.8%) LOs were not rated due to a lack of expert knowledge.

**Figure 2:**
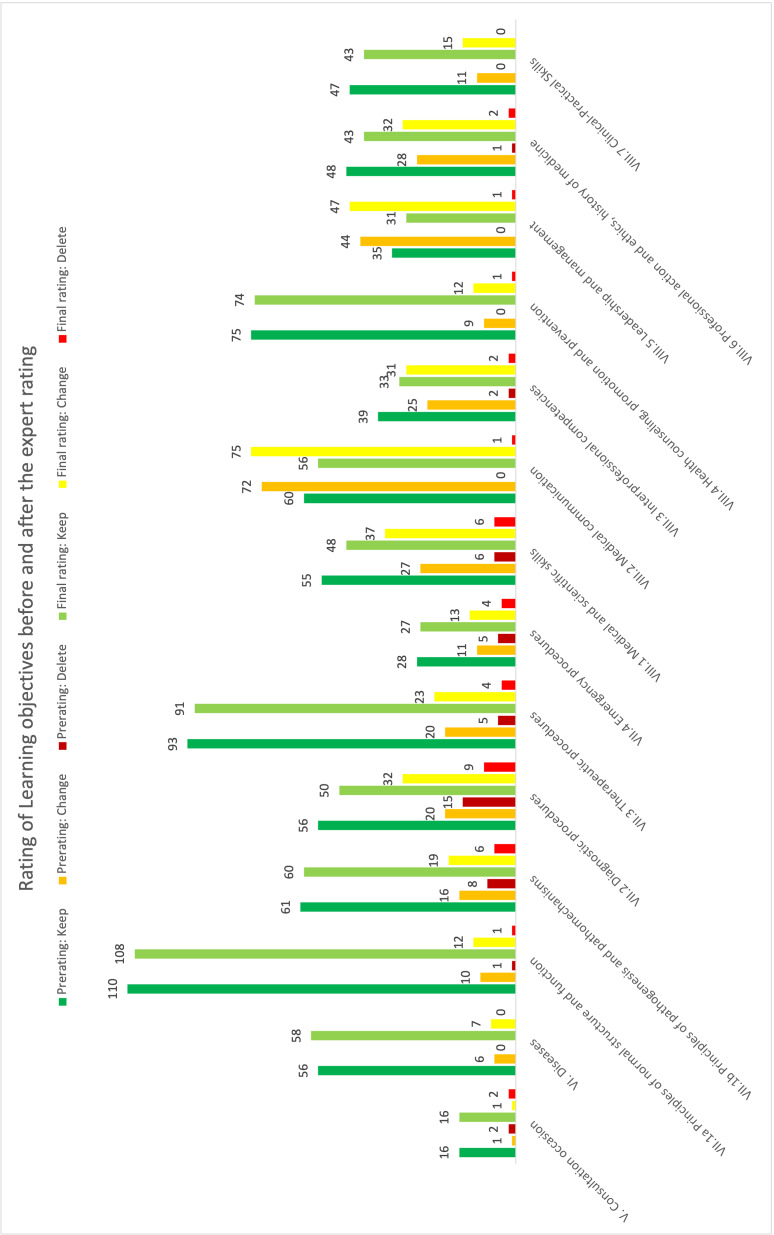
Rating of the Learning objectives of phases 1 and 2 before and after the expert rating per NKLM chapter

In the third step, the 264 new LOs and the 45 LOs with pre-rating “delete” were discussed within a group of medical experts and students. The pre-rating was changed in 72 cases, see table 2 (Tab. 2) and figure 2 (Fig. 2) for detailed information.

Finally, 738 LOs were rated to be kept, 356 to be modified, and 39 to be deleted (see table 3 (Tab. 3)). Overall, the percentage of the ratings “keep”, “modify” and “delete” differed between the chapters (see table 2 (Tab. 2) and table 3 (Tab. 3)).

During the evaluation process, several general issues came up. First, the wording was not consistent in terms of gender-sensitive language. It was unclear how to handle LOs with similar topics but in different chapters (e.g., consultation and diseases). Redundancies were often encountered, but they were distributed across different chapters. Identifying these LOs takes time but reduction of redundancy is crucial to reduce the extent of NKLM. Another issue was that several new LOs based on the same old LO were not in the same rating phase, making it difficult for faculties to comprehend the modifications. Finally, there were no recommendations for teaching LOs from chapters VII.01 and VII.02 in higher semesters (milestones with competency level per semester). This is particularly relevant for study programs where the theoretical and clinical parts of the curriculum are closely interconnected (“Modellstudiengänge”), as selected basic LOs can be taught in higher semesters.

## Discussion

In this article, we reported on the evaluation process of Learning Objectives from the National Competence-Based Catalogue for Undergraduate Medical Education (NKLM) at Hannover Medical School. The evaluation process consisted of three steps: comparison of the mapping of the NKLM 1.0 in Hannover with the new learning objectives (LOs) of NKLM 2.0, a pre-rating conducted by the persons responsible for curricular development, and finally a discussion in a team of medical experts and students to reach a consensus on the rating. The process was performed within 2,5 personnel months. Ultimately, 738 LOs were rated to be kept, 356 to be modified, and 39 to be deleted.

The evaluation process faced several challenges. In a previous study at Hannover Medical School, 30% of the medical educators stated that they were not familiar with the catalogue [6]. Those persons will encounter difficulties in finding a valid rating for differences between the old and the new NKLM. For this reason, we decided to focus on the text of the LOs and not on the even more complex interconnections. Previous studies from southern Germany reported that faculty members were not per se willing to deal with the NKLM due to its complexity, but supportive administrational structure was favorable for motivation to take part in the rating process [8], [9].

To address these issues, the complete process was conceived comprising three steps. To increase willingness of faculty experts to participate in the rating process, a time-consuming pre-rating was established. In this pre-rating redundancies were identified and omitted. Additionally, issues related to wording were identified and changed if necessary. This pre-rating-based clearing up allowed the expert und student teams to focus on specific content aspects. In fact, the 18 medical experts and five students discussed and consensually decided on keeping, modifying and deleting in a way not distracted by purely formal questions. The chapters of the NKLM 2.0 were written by different authors with differing levels of details in the LOs. In our process, we noticed different percentages of the ratings for the chapters. Our rating included for example to summarize many LOs in chapter VII.2 since each LO described a very specific diagnostic procedure. In the NKLM 1.0, these procedures were condensed, making the NKLM more appropriable. This underlines the importance of an overall review process, to adapt a comparable level of details for each chapter. 

The strengths of our performed evaluation process include

1. structured organization of the process,
2. effective time management,
3. clearing up of formal questions and
4. the consideration of both overview knowledge and expert knowledge.

The pre-rating allowed a more effective and result-oriented discussion. Inclusion of students in this rating-process was in line with our previous positive experiences [10]. Students are not experts per se but they gave a new perception of some aspects.

Overall, the MFTs’ goal of a significant decrease of LOs was not met. This could be the case because the single LOs are difficult to rate, if similar LOs are not in the same rating phase. Also, an overview of interconnections could lead to a reduction of LOs. 

Still, our experiences can serve as a basis for the upcoming evaluation process of phases 3 and 4 of the NKLM, especially for faculties with time-intense rating processes. Therefore, a focus on the reduction of LOs and interconnections might be necessary. Also In an international context, catalogues with learning objectives gain more importance. The description of our rating process can be adapted for similar developments in different countries. 

## Conclusion

In conclusion, our evaluation process of LOs of NKLM 2.0 was based on a structured procedure allowing the effective and motivated involvement the faculty experts. Prepared by experts familiar with both, the NKLM and the overall medical curriculum, the process allowed detection of redundancies and the identification of LOs within the current curriculum. Medical faculty experts and students became also involved in the evaluation process, ensuring a high quality of the rating-process. 

## Acknowledgements

Special thanks to Sandra Friesen for her support in documentation and preparation of the process. 

## Competing interests

The authors declare that they have no competing interests. 

## Figures and Tables

**Table 1:**
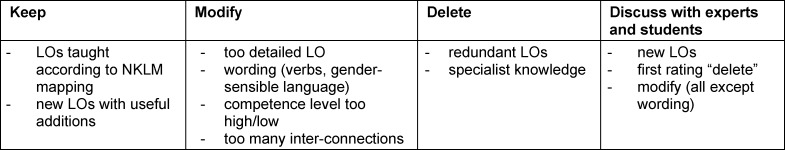
Criteria for the evaluation of NKLM Learning objectives

**Table 2:**
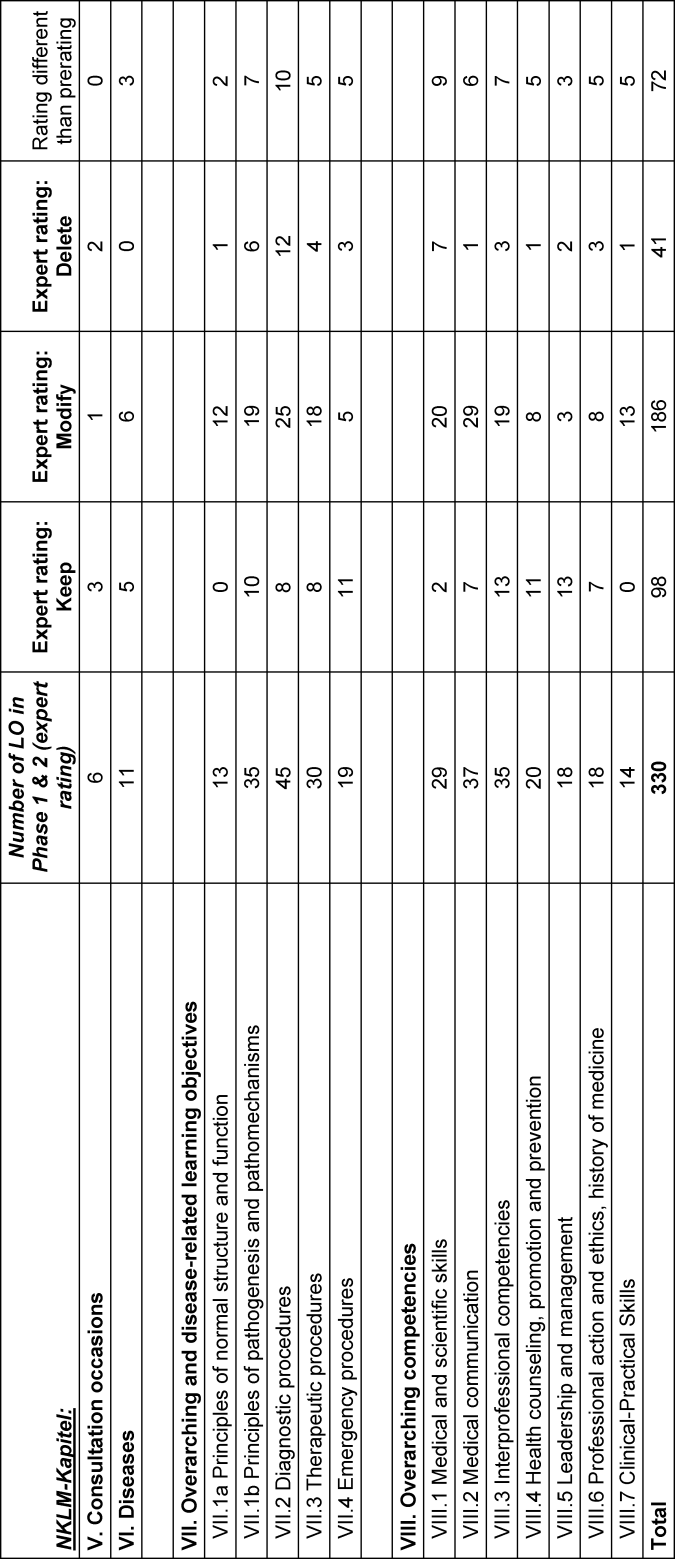
Expert rating of the Learning objectives per NKLM chapter

**Table 3:**
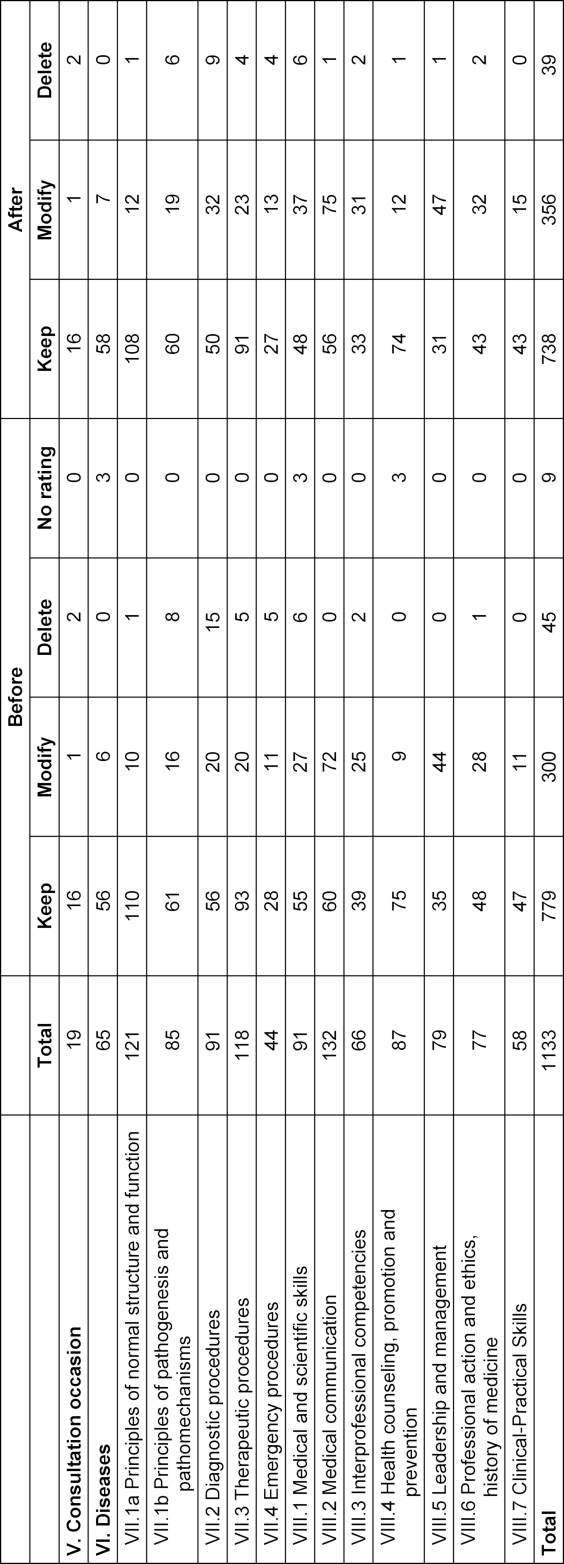
Rating of learning objectives of the phases 1 and 2 of NKLM 2.0 before and after Expert rating per chapter
